# Dynamic risk assessment of a coal slurry preparation system based on the structure-variable Dynamic Bayesian Network

**DOI:** 10.1371/journal.pone.0302044

**Published:** 2024-05-21

**Authors:** Ming Liu, Liping Wu, Mingjun Hou

**Affiliations:** 1 School of Environment and Safety Engineering, Liaoning Petrochemical University, Fushun, Liaoning, China; 2 General graduate school, Woosuk University, Wanju-Gun, Jeollabuk-do, South Korea; Tribhuvan University, NEPAL

## Abstract

In order to strengthen the safety management of coal slurry preparation systems, a dynamic risk assessment method was established by using the bow-tie (BT) model and the Structure-variable Dynamic Bayesian Network (SVDBN). First, the BT model was transformed into a static Bayesian network (BN) model of the failure of a coal slurry preparation system by using the bow-tie model and the structural similarity of the Bayesian cognitive science, based on the SVDBN recursive reasoning algorithm. The risk factors of the coal slurry preparation system were deduced using the Python language in two ways, and at the same time, preventive measures were put forward according to the weak links. In order to verify the accuracy and feasibility of this method, the simulation results were compared with those obtained using GeNIe software. The reasoning results of the two methods were very similar. Without considering maintenance factors, the failure rate of the coal slurry preparation system gradually increases with increasing time. When considering maintenance factors, the reliability of the coal slurry preparation system will gradually be maintained at a certain threshold, and the maintenance factors will increase the reliability of the system. The proposed method can provide a theoretical basis for the risk assessment and safety management of coal slurry preparation systems.

## Introduction

The current situation of an oil shortage, a gas shortage, and relatively abundant coal resources has led to the dominant position of China’s coal energy. The development of modern technology in the coal chemical industry is necessary to meet national strategic needs [1]. Coal gasification plays an important role in production in the coal chemical industry. Coal gasification is characterized by release of many flammable, explosive, toxic, harmful, and corrosive substances, and it is a complex, large-scale, and high-density process. Accidents cause serious damage to people, property, and the environment [2–4]. The characteristics and inherent risks of coal chemical production determine the importance of risk assessment in the coal chemical industry. At present, most risk assessments in the field are static risk assessments. If the time factor can be considered, the assessment results can be much more accurate, which is of great significance for the risk assessment of the coal chemical process.

**Fig 1:**
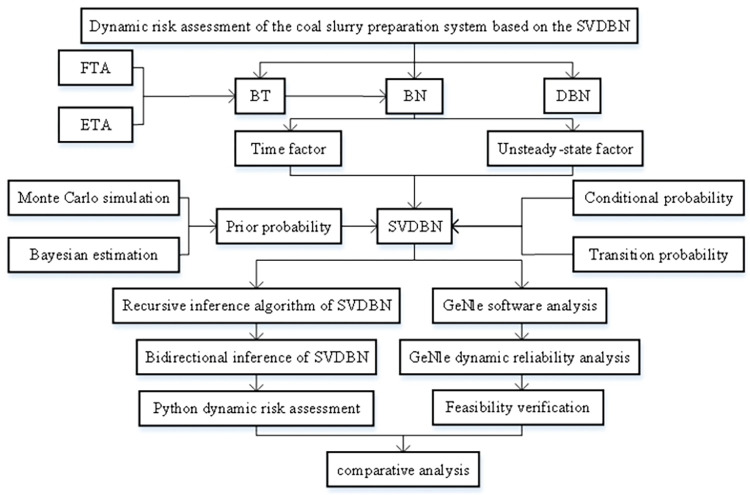
Risk assessment process.

Current research on the risk analysis of coal gasification plants is mainly based on traditional risk analysis methods, such as fault tree analysis (FTA), event tree analysis (ETA), and hazard and operability analysis (HAZOP). It focuses on identifying risk factors in the technological process and qualitatively analyzing the causes and consequences of a deviation from the process. Few studies have used quantitative dynamic risk analysis methods. In recent years, BN has been widely used in the quantitative assessment of the risks of the petrochemical industry, due to its powerful probabilistic reasoning ability [5–7]. Sun [8] proposed a risk management and control model for the coal gasification plant, selected key dangerous events to establish a BT model and evaluate the performance of relevant safety barriers, and used a BN to determine the main risk factors influencing the plant. Laal [9] used trapezoidal fuzzy numbers to calculate the failure rates and transferred them into a Bayesian network (BN) for risk analysis using RoV in GeNle software. Pouyakian [10] proposed a fuzzy Bayesian network (FBN) approach to analyze the domino effects of pool fire. Hanifi [11] used Bayesian networks (BNs) to update the speed with which fire spread. Khoshakhlagh [12] presented a holistic model based on the Fuzzy Bayesian Network–Human Factor Analysis and Classification System (FBN-HFACS) to analyze the factors in the COVID-19 pandemic that related to risk management under uncertainty. Mohammadi [13] used an integrated approach including BT, fuzzy Bayesian networks, and consequence modeling to estimate risk in tank storage. Jabbari [14] presented a risk assessment method based on a fuzzy Bayesian network (FBN) and the William Fine method in low-voltage power distribution systems. However, the above research methods all consider risk variables in a static way, without considering dynamic risk factors such as equipment aging, human error, and seasonal change. In view of BN’s ability to adapt to probability updates, Khakzad [15]. proposed a dynamic risk identification method that maps the dangerous scene of the process system to BN. However, BN is still limited to reflecting the dynamic evolution between real-time faults, and the Markov process is introduced into BN, which can deal with the state transition of time-dependent variables. The DBN-based dynamic risk assessment model is generally transformed from the traditional risk assessment model and combines the dynamic characteristics of human factors, common cause failures, and degradation processes for risk assessment [16–19]. Wu [20] and Chang [21] used DBN to predict and diagnose offshore drilling accidents and leakage accidents at hydrogen production units, which can be quantitatively inferred on different time slices. The dynamic Bayesian network (DBN) structure model can effectively represent the structural relationship between node variables in the dynamic risk assessment system of the coal chemical industry and can also calculate the exact value of risk, so its use as the main research method for the dynamic risk assessment of the coal gasification process is suitable. Based on DBN, Liu [22] conducted a dynamic risk assessment of the changes in the reliability of a gasifier burner system during the operating cycle. The dynamic reliability of the system was inferred from prior data; it was found that the dynamic reliability of the system and its subsystems gradually decreased with an increase in operating time, and the weak links of the system were successfully identified. In addition, Liu [23] proposed an evaluation method that combined the cloud model with a DBN, conducted a comprehensive analysis of importance, and completed risk prediction and evaluation for the gasifier system. However, maintenance factors were not considered in this research. In practice, maintenance factors also play a vital role in reducing the probability of accidents. Gao [24] conducted a DBN risk assessment for the over-temperature failure of the gasifier system. Considering the influence of maintenance factors on the over-temperature of the system, it was found that the maintenance factors had little effect in the early stages of operation, but played a great role in reducing the probability of accidents over time. In addition, the operation process of the coal chemical industry is not only time-varying but also unstable. The SVDBN is more suitable, flexible, and effective than the traditional dynamic Bayesian network. The coal slurry preparation system is the initial link in the coal gasification process. During the operation of the system, the energy consumption and material consumption are constantly concentrated and expanded, which means that an accident can have serious consequences. Therefore, this study took a coal slurry preparation system as the research object and constructed a static Bayesian network (BN) structure model based on the bow-tie model (BT) considering the causal correlation. Since the traditional dynamic Bayesian network is based on the steady-state hypothesis, but the change in the coal chemical operation process is not stable, a structure-variable dynamic Bayesian network (SVDBN) was adopted to carry out dynamic risk assessment on the fault of a coal slurry preparation system in an unsteady state. The influences of time variability and maintainability on the system dynamic risk assessment were fully considered, and the dynamic failure rate of the system was obtained through forward reasoning. The weak link was found by backward reasoning. The specific risk assessment process is shown in Fig 1.

## Risk analysis method

### Bow-tie model analysis

**Fig 2:**
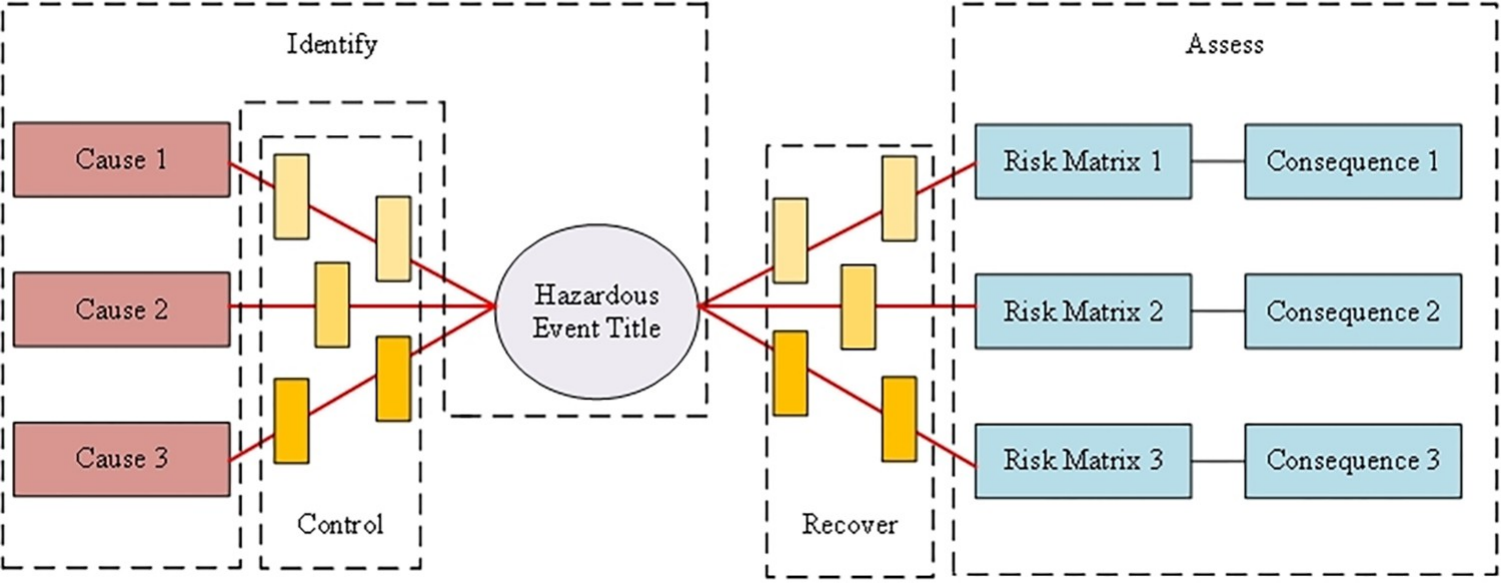
BT model.

The BT model is a comprehensive risk analysis method that combines a fault tree and an event tree. It is widely used in safety evaluation, as it intuitively and comprehensively analyzes several elements that lead to top events, and enables the effective prevention of accidents by allowing sources of risk to be identified, risk factors to be discriminated, safety barriers to be set, and control measures to be implemented to prevent and reduce risks. Among the many accident analysis models, the BT model has been shown to be reliable for accident risk assessment and risk management [25, 26]. A typical BT model is shown in Fig 2, where the fault tree is on the left and the event tree is on the right. When the safety barrier in the picture is broken, a dangerous event escalates. If recovery measures are not in place, the dangerous event escalates into the undesirable consequences of an accident.

The analysis steps for the BT model are as follows: (1) identify potentially dangerous and harmful factors; (2) determine the top event, find the cause of the top event, and perform a fault tree analysis; (3) analyze the different consequences resulting from different causes of the top event, and perform an event tree analysis; (4) analyze the root cause of the accident and take preventive measures; (5) present effective measures to reduce the consequences of the accident [27].

### Transformation from BT model to BN model

The transformation from the BT model to a BN includes structural transformation and static logic gate parameter transformation. The transformation rules are as follows:

(1) Structural transformation

**Fig 3:**
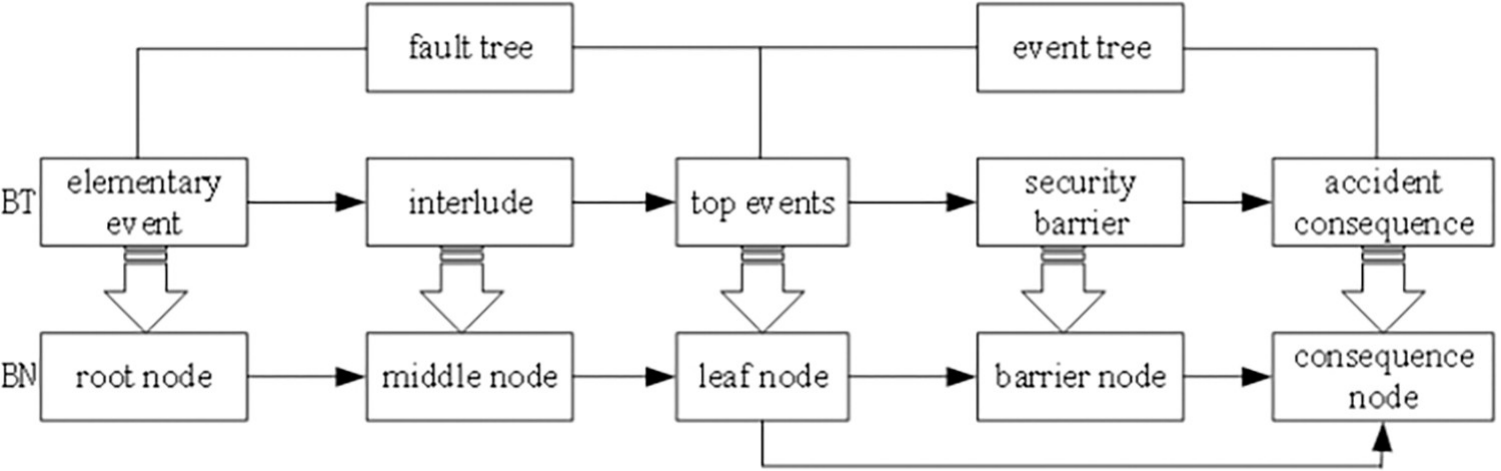
Transformation of BT model into BN model.

The flow of the BT model transformation into a BN model is shown in Fig 3.

(2) Parameter transformation of static logic gates

BNs converted from the AND gate and OR gate are the same in structure, but their conditional probabilities are different. Let T = 0 mean that the event does not occur and T = 1 mean that the event occurs. The conditional probabilities are shown in Eq (1) and Eq (2), respectively.


$$\left\{ \begin{array}{l} P(T=1|A=0,B=0)=0\\ P(T=1|A=0,B=1)=0\\ P(T=1|A=1,B=0)=0\\ P(T=1|A=1,B=1)=1 \end{array}\right.$$ (1)

$$\left\{ \begin{array}{l} P(T=1|A=0,B=0)=0\\ P(T=1|A=0,B=1)=1\\ P(T=1|A=1,B=0)=1\\ P(T=1|A=1,B=1)=1 \end{array}\right.$$ (2)

### Structure-variable Dynamic Bayesian Network method

**Fig 4:**
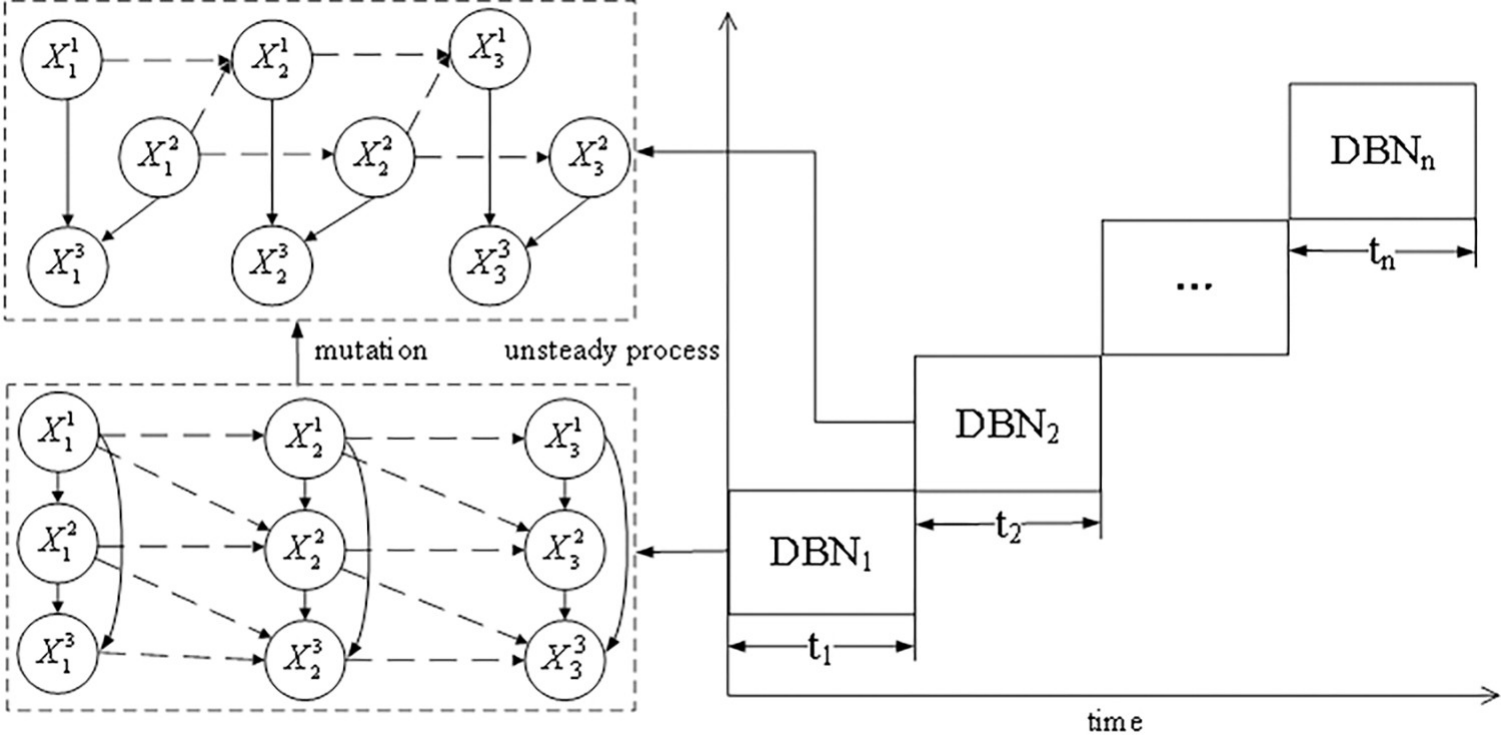
SVDBN schematic diagram of the unsteady state process.

The dynamic risk assessment of a coal slurry preparation system was performed using the SVDBN in an unsteady state. The SVDBN can be regarded as composed of several different DBNs (S1 File). The structure of the DBN of each time slice is different, and the transfer network between adjacent DBNs is also different [28–30]. Its principle is shown in Fig 4.

For the SVDBN with T time slices, B^1^ is the initial network and P(Z_1_) is the initial time probability. $B_{\to }^{t}$ is the BN of two adjacent time slices, and the conditional probability of the transition network is:

$$P(Z_{t}|Z_{t-1})=\prod_{t=1}^{n_{t}}P(Z_{t}^{i}|Pa(Z_{t}^{i}))$$ (3)

where *n*_*t*_ is the number of nodes in the t-th time slice.

The conditions for constructing an SVDBN model with T time slices are as follows: a DBN graph of time slices T and a conditional probability table of the BN of each time slice. InterCPT_1_, InterCPT_2_, InterCPT_T_; InterCPT_1_, InterCPT_2_,…, InterCPT_T-1_ are conditional probability tables for T-1 transition networks representing BN dependencies of adjacent time slices.

**Fig 5:**
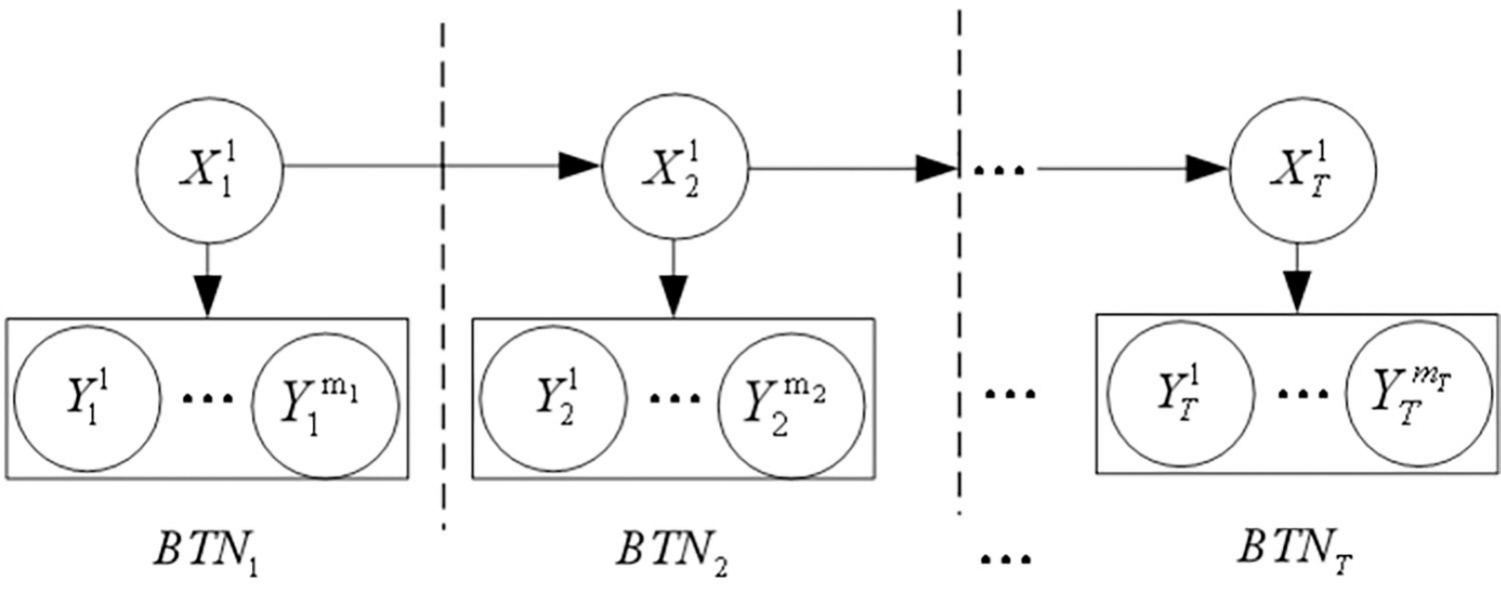
SVDBN recursive inference algorithm network diagram.

Suppose that SVDBN has T time slices; the BN structure of the T time slice is BN_t_. There is a hidden node and there are m_t_ observation nodes; the hidden node is represented by X_t_, and there are n_t_ states, namely, {1, 2,…, n}; $Y_{t}^{v}(v=1,2,\ldots ,m_{t})$ is used to represent the observed variable v of the t-th time slice, which is only dependent on other variables in this time slice, and its observed value is $y_{t}^{v}$. It is assumed that the observation data on the t-th time slice are $y_{t}^{1:m_{t}}=\{y_{t}^{1},y_{t}^{2},\cdots y_{t}^{m_{t}}\}$, and the conditional probability table of the t-th time slice is IntraCPT_t_. The SVDBN can be inferred using a recursive inference algorithm. Its network diagram is shown in Fig 5.

If we define the forward operator of the t-th time slice as *α*_t_(*i*), then $\alpha_{t}(i)=P(X_{t}=i|y_{1}^{1\cdot m_{1}},y_{2}^{11m_{2}},\cdots ,y_{t}^{1.m_{t}})$, where *i* indicates the state of the observation node *X*_*t*_, and the value is 1,2,…,*S*_*t*_; $y_{t}^{1:m_{t}}$ is the observation node state on the t-th time slice.

(1) Recursive forward process of SVDBN

Forward inference initialization: $\alpha_{1}(i)=\eta \pi (i)\prod_{v=1}^{m_{1}}P(y_{1}^{v}|Pa(Y_{1}^{v}))$, where $\pi (i)=P(X_{1}=i)$ is an a priori probability; $\sum_{i=1}^{n_{1}}\pi (i)=1$; $Pa(Y_{1}^{v})$ is the set of parent nodes of the observation node $Y_{1}^{v}$ on the first time slice; and *η* is the normalization factor.

The iterative calculation of forward reasoning is as follows:

$$\begin{array}{l} \alpha_{t}(j)=P(X_{t}=j|y_{1}^{1:m_{1}},y_{2}^{1:m_{2}},\ldots ,y_{t}^{1:m_{t}})\\ =\eta \prod_{v=1}^{m_{t}}P(y_{t}^{v}|Pa(Y_{t}^{v}))\sum_{i=1}^{n_{t-1}}\alpha_{t-1}^{ij}•P(X_{t-1}=i|y_{1}^{1:m_{1}},y_{2}^{1:m_{2}},\ldots ,y_{t-1}^{1:m_{t-1}})\\ =\eta \prod_{v=1}^{m_{t}}P(y_{t}^{v}|Pa(Y_{t}^{v}))\sum_{i=1}^{n_{t-1}}\alpha_{t-1}^{ij}\alpha_{t-1}(i) \end{array}$$ (4)

where *j* = 1,2,…,*n*_*t*_, t = 1, 2,…, T.

(2) Recursive backward process of SVDBN

Define the t-th time slice backward operator as *β*_*t*_(*i*), then $\beta_{t}(i)=P(y_{t+1}^{1:m_{t+1}},y_{t+2}^{1:m_{t+2}},\ldots ,y_{T}^{1:m_{T}}|X_{t}=i)$ where *i* = 1,2,…,*n*_*t*_ t = 1, 2,…, T.

Backward inference initialization: *β*_*T*_(*i*) = 1.

Iterative calculation of backward reasoning:

$$\begin{array}{l} \beta_{t}(i)=P(y_{t+1}^{1:m_{t+1}},y_{t+2}^{1:m_{t+2}},\ldots ,y_{T}^{1:m_{T}}|X_{t}=i)\\ =\sum_{j=1}^{n_{t+1}}P(y_{t+2}^{1:m_{t+2}},\ldots ,y_{T}^{1:m_{T}},X_{t+1},=j,y_{t+1}^{1:m_{t+1}}|X_{t}=i)\\ =\sum_{j=1}^{n_{t+1}}P(y_{t+2}^{1:m_{t+2}},\ldots ,y_{T}^{1:m_{T}}|X_{t+1}=j)P(X_{t+1}=j{|X}_{t}=i)•\prod_{v=1}^{m_{t+1}}P(y_{t+1}^{v}|Pa(Y_{t+1}^{v}))\\ =\sum_{j=1}^{n_{t+1}}\beta_{t+1}(j)\alpha_{t}^{ij}\prod_{v=1}^{m_{t+1}}P(y_{t+1}^{v}|Pa(Y_{t+1}^{v})) \end{array}$$ (5)

where $Pa(Y_{t+1}^{v})$ is the collection of parent nodes of observation nodes $Y_{t+1}^{v}$ on the (t+1)-th time slice.

By synthesizing the forward algorithm and backward algorithm, the SVDBN recursive reasoning algorithm can be obtained:

$$\begin{array}{l} \gamma_{t}(i)=P(X_{t}=i|y_{1}^{1:m_{1}},y_{2}^{1:m_{2}},\cdots ,y_{T}^{1:m_{T}})\\ =P(X_{t}=i|y_{1}^{1:m_{1}},y_{2}^{1:m_{2}},\cdots ,y_{t}^{1:m_{t}},y_{t+1}^{1:m_{t+1}},\cdots ,y_{T}^{1:m_{T}})\\ =\eta P(X_{t}=i|y_{1}^{1:m_{1}},y_{2}^{1:m_{2}},\cdots ,y_{t}^{1:m_{t}})P(y_{t}^{1:m_{t}},y_{t+1}^{1:m_{t+1}},\cdots ,y_{T}^{1:m_{T}}|X_{t}=i,y_{1}^{1:m_{1}},y_{2}^{1:m_{2}},\cdots ,y_{t}^{1:m_{t}})\\ =\eta P(X_{t}=i|y_{1}^{1:m_{1}},y_{2}^{1:m_{2}},\cdots ,y_{t}^{1:m_{t}})P(y_{t}^{1:m_{t}},y_{t+1}^{1:m_{t+1}},\cdots ,y_{T}^{1:m_{T}}|X_{t}=i)\\ =\eta \alpha_{t}(i)\beta_{t}(i) \end{array}$$ (6)

**Fig 6:**
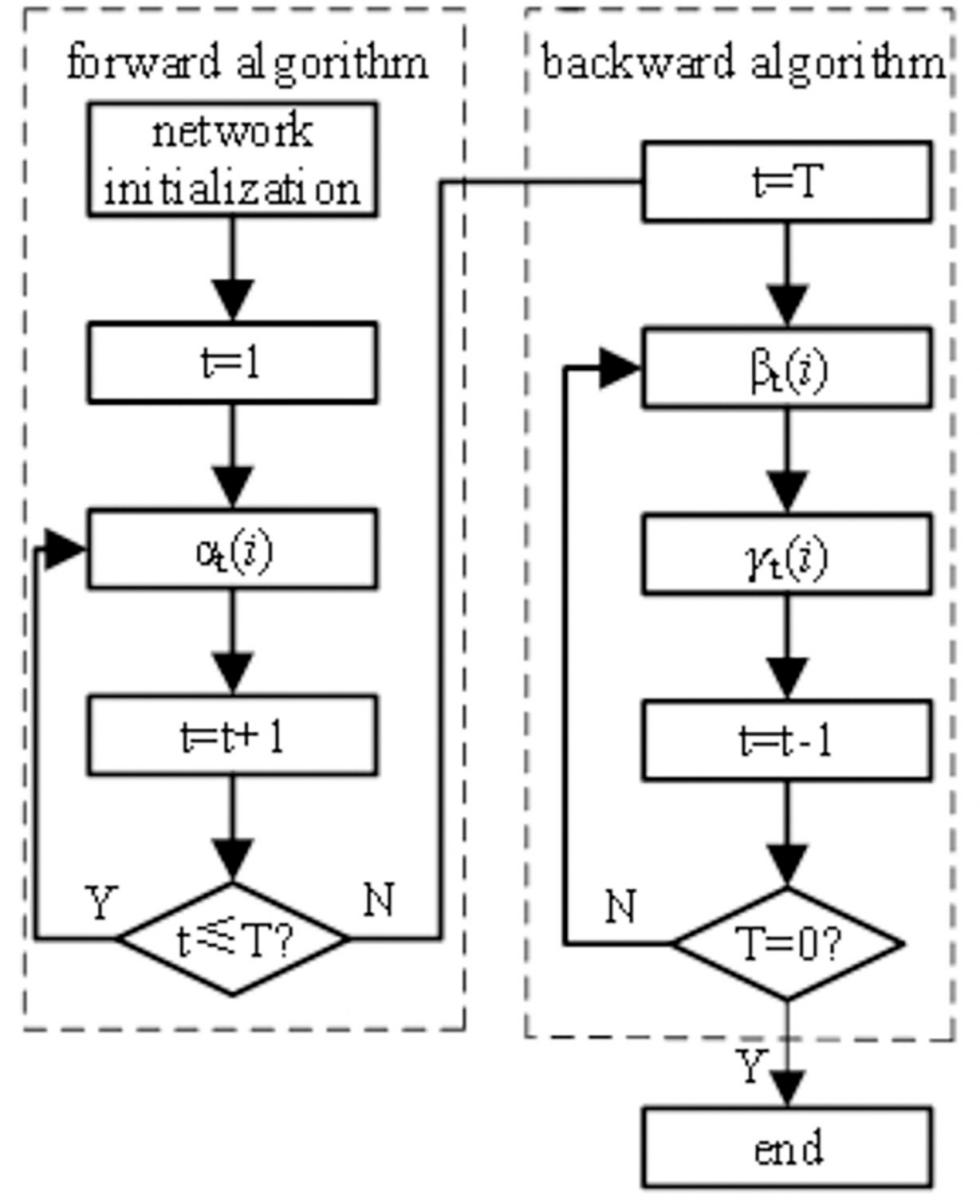
Flow chart of the recursive reasoning algorithm in SVDBN.

The flow of the SVDBN recursive reasoning algorithm is shown in Fig 6.

In this study, the SVDBN model of the coal slurry preparation system was unchanged in the same time slice and between different time slices, and what changes with time is the conditional probability of the root node across different time slices.

## Coal slurry preparation system

### Process flow of the coal slurry preparation system

**Fig 7:**
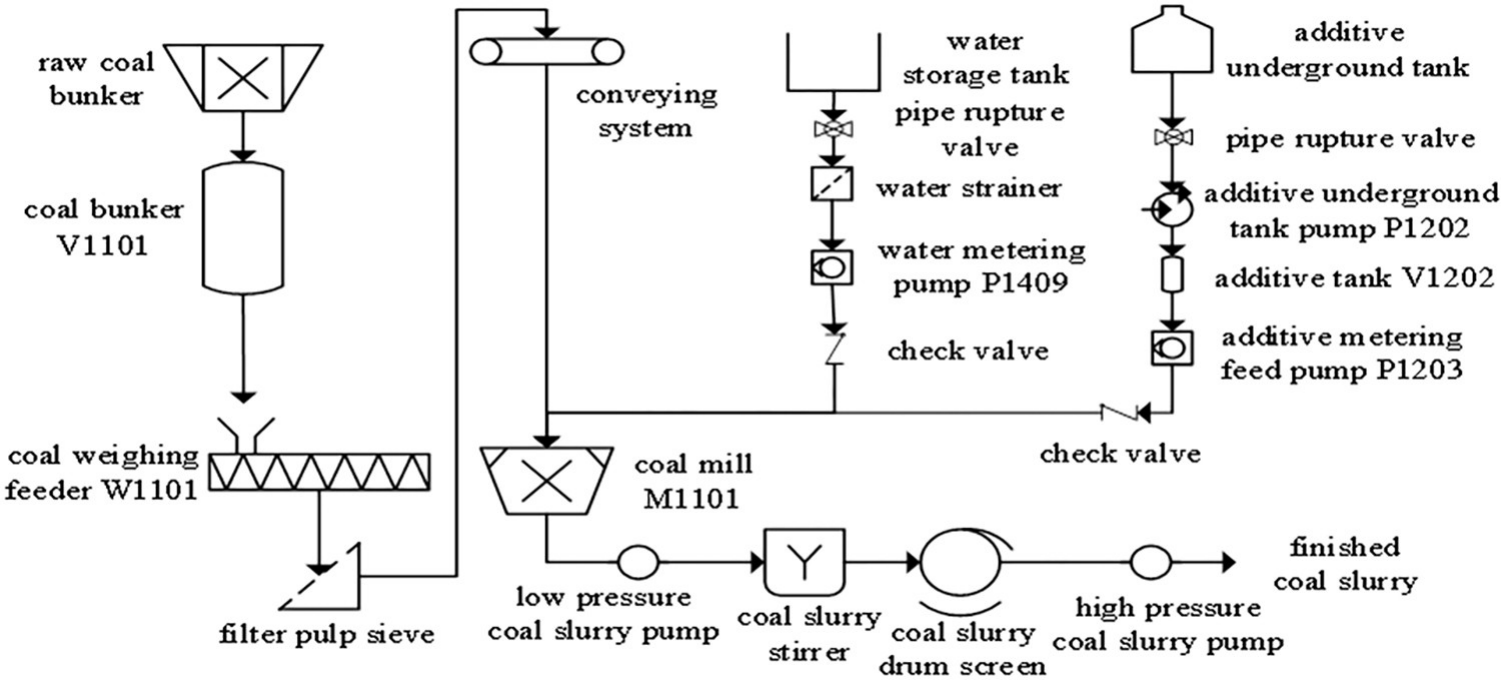
Coal slurry process flow diagram.

The coal slurry preparation process involves the transport of the coal stored in the raw coal bunker V1101 to the coal mill M1101 through the coal weighing feeder W1101 for coal grinding, and the conveyance of the additive stored in the additive underground tank to the coal mill M1101 through the additive feeding pump P1203. At the same time, the required water is transported to the coal mill M1101 through the water metering pump P1409 to make crude coal water slurry. The crude coal water slurry enters the coal slurry mixer and coal slurry drum screen from the low-pressure coal slurry pump, and then enters the high-pressure coal slurry pump to make the finished coal slurry. The process flow is shown in Fig 7.

Through investigation and analysis, it was found that the causes of the failure of the coal slurry system include an abnormal water supply in the coal mill, an abnormal coal flow rate, an abnormal additive flow rate, a low liquid level of V1102 in the discharge tank of the mill, a failure of the auxiliary system, etc. A failure results in the mill system shutting down, mill slurry running, vehicle jumping, and the concentration and viscosity of the coal slurry becoming abnormal.

### BT model of the coal slurry preparation system

**Fig 8:**
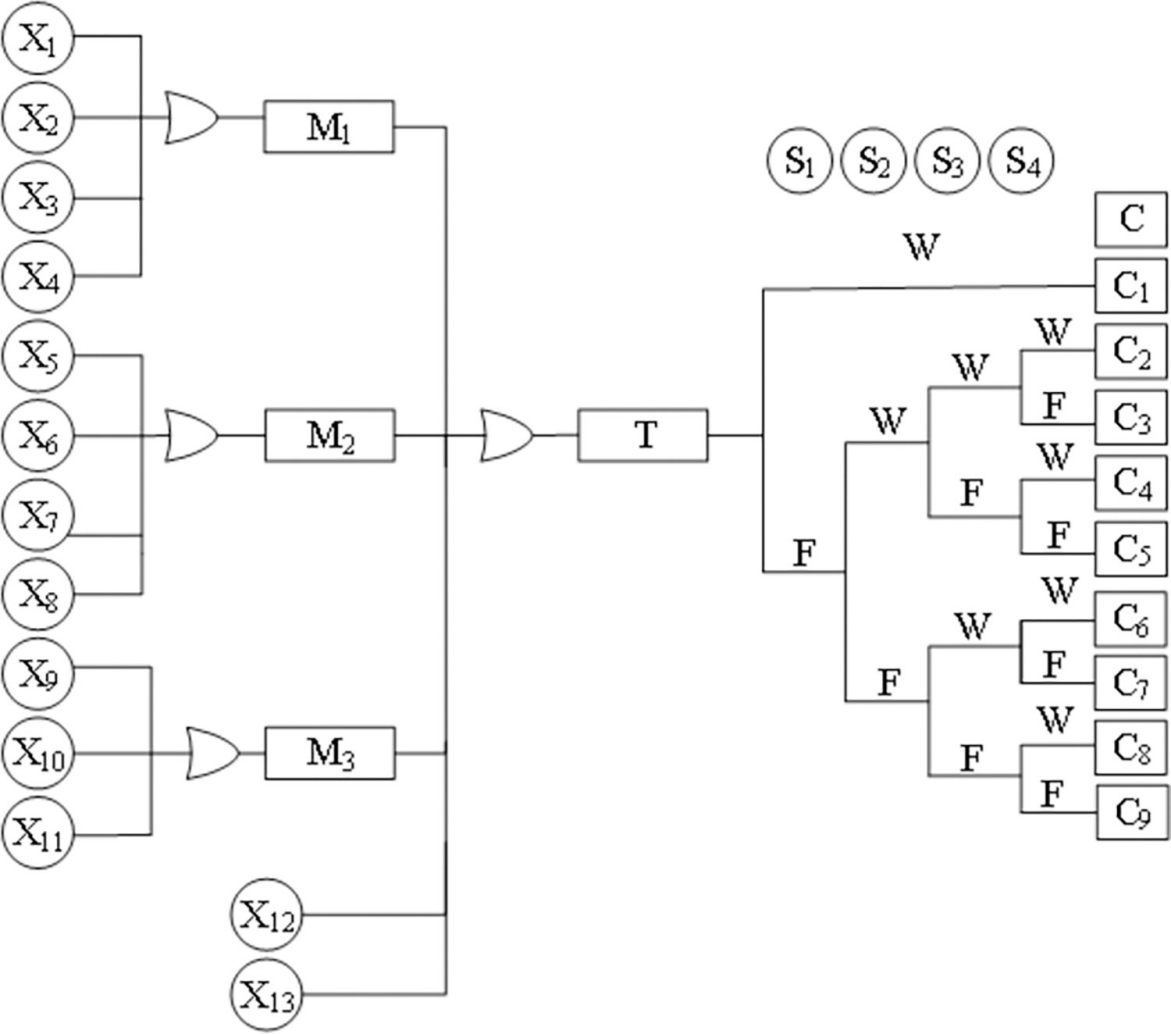
BT model of the coal slurry preparation system.

The BT model of the coal slurry preparation system was constructed according to the process flow, as shown in Fig 8.

The names of each node are shown in Table 1.

**Table 1:** Node number and name.

Node	Event name	Node	Event name	Node	Event name
T	Coal slurry preparation system failure	X_7_	Coal bunker level indicator WI1101 fault	S_4_	Additive flowmeter FI1204 failure
M_1_	Abnormal water supply of coal mill	X_8_	Coal bunker level indicator WI1101 control loop failure	C_1_	System security, smooth operation
M_2_	Abnormal coal flow	X_9_	Additive metering pump P1203 fault	C_2_	System operates safely based on barrier protection.
M_3_	Abnormal flow of additives	X_10_	Additive metering pump P1203 inlet filter blockage	C_3_	Mill run slurry, coal slurry viscosity is abnormal
X_1_	Additive feed pump P1203 abnormal measurement	X_11_	Additive flowmeter FI1204 failure	C_4_	Mill run slurry, jumps, coal slurry concentration is abnormal.
X_2_	Additive feed pump P1203 inlet pipeline blocked	X_12_	Mill discharge tank V1102 liquid level is too low	C_5_	Mill run slurry, jumps, and the coal slurry concentration and viscosity are abnormal.
X_3_	Flow controller FIC1101 valve circuit fault	X_13_	Auxiliary system failure	C_6_	Mill system shutdown
X_4_	FI1101A/B water flow meter shows low	S_1_	Personnel did not find and deal with in time	C_7_	Mill system shutdown, run slurry, coal slurry viscosity is abnormal
X_5_	Coal bunker V1101 empty bunker	S_2_	Coal mill system shutdown interlock failure	C_8_	Mill system shutdown, run slurry, jump, coal slurry concentration is abnormal
X_6_	Coal bunker V1101 coal bridge	S_3_	Feedwater flowmeter FI1101 failure	C_9_	Mill system shutdown, run slurry, jump, coal slurry concentration and viscosity are abnormal.

Because the coal slurry preparation system has a short running time and no reference to failure data, the failure rate of each event node is obtained by consulting the literature, combining Bayesian estimation [31] and Monte Carlo simulation, as shown in Table 2:

**Table 2:** Node failure rate.

Node	Failure rate λ	Node	Failure rate λ	Node	Failure rate λ
X_1_	7.92E-5	X_7_	8.93 E-5	X_13_	1.48E-5
X_2_	8.69E-5	X_8_	6.13 E-5	S_1_	1.31 E-4
X_3_	3.44E-4	X_9_	1.16E-4	S_2_	6.88 E-4
X_4_	5.35 E-5	X_10_	2.56 E-5	S_3_	6.21 E-5
X_5_	8.46E-5	X_11_	1.69E-4	S_4_	2.59 E-4
X_6_	1.78 E-4	X_12_	2.98E-4		

### Bayesian network model of the coal slurry preparation system

**Fig 9:**
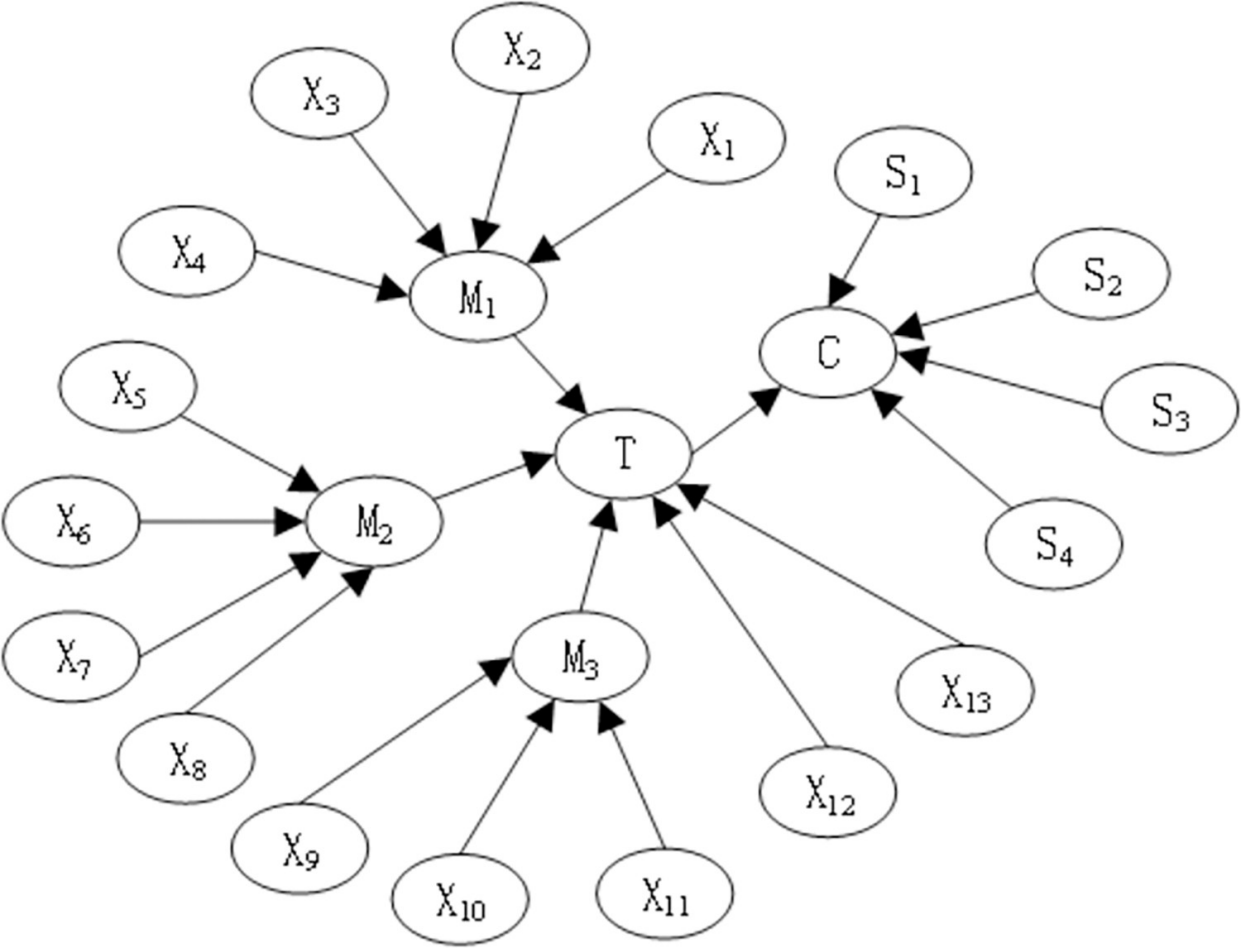
BN model of coal slurry preparation system.

The BN model of the coal slurry preparation system can be obtained according to the principle of conversion from BT to BN, as shown in Fig 9.

The network structure and conditional probability table in the time slice of the SVDBN were converted from the BN. The conditional probability table between the nodes that span time slices was obtained from the fault probability density function of the nodes. Assuming that the fault probability density function of node A is f_A_ (t) and that the system can be repaired without considering the fault factors, the failure probability of dynamic nodes obeys the exponential distribution, from which the conditional transition probability from time t to time *t*+Δ*t* can be obtained as follows:

$$\left\{ \begin{array}{l} P(A(t+\Delta t)=0|A(t)=0)=e^{-\lambda t}\\ P(A(t+\Delta t)=1|A(t)=0)=1-e^{-\lambda t}\\ P(A(t+\Delta t)=1|A(t)=1)=1\\ P(A(t+\Delta t)=0|A(t)=1)=0 \end{array}\right.$$ (7)

Let Δ*t* = 400*h*; the SVDBN model of the coal slurry preparation system running for 2000 h was constructed, and there were five time slices. The conditional probability between the time slices of each node is shown in Table 3:

**Table 3:** Conditional probability of time slice between nodes in coal slurry preparation system.

Node	Time slice 1	Time slice 2	Time slice 3	Time slice 4	Time slice 5
X_1_	0.0312	0.0316	0.0320	0.0323	0.0327
X_2_	0.0342	0.0346	0.0350	0.0354	0.0358
X_3_	0.1286	0.1301	0.1315	0.1330	0.1345
X_4_	0.0212	0.0214	0.0217	0.0220	0.0222
X_5_	0.0333	0.0337	0.0341	0.0345	0.0349
X_6_	0.0687	0.0696	0.0704	0.0712	0.0720
X_7_	0.0351	0.0355	0.0360	0.0364	0.0368
X_8_	0.0242	0.0245	0.0248	0.0251	0.0254
X_9_	0.0453	0.0459	0.0464	0.0470	0.0476
X_10_	0.0102	0.0103	0.0104	0.0106	0.0107
X_11_	0.0654	0.0662	0.0669	0.0677	0.0685
X_12_	0.1124	0.1137	0.1150	0.1163	0.1176
X_13_	0.0059	0.0060	0.0061	0.0061	0.0062
S_1_	0.0511	0.0517	0.0523	0.0529	0.0535
S_2_	0.2406	0.2432	0.2458	0.2484	0.2510
S_3_	0.0245	0.0248	0.0251	0.0254	0.0257
S_4_	0.0984	0.0996	0.1007	0.1019	0.1031

## Discussion

### SVDBN prediction of the coal slurry preparation system

**Fig 10:**
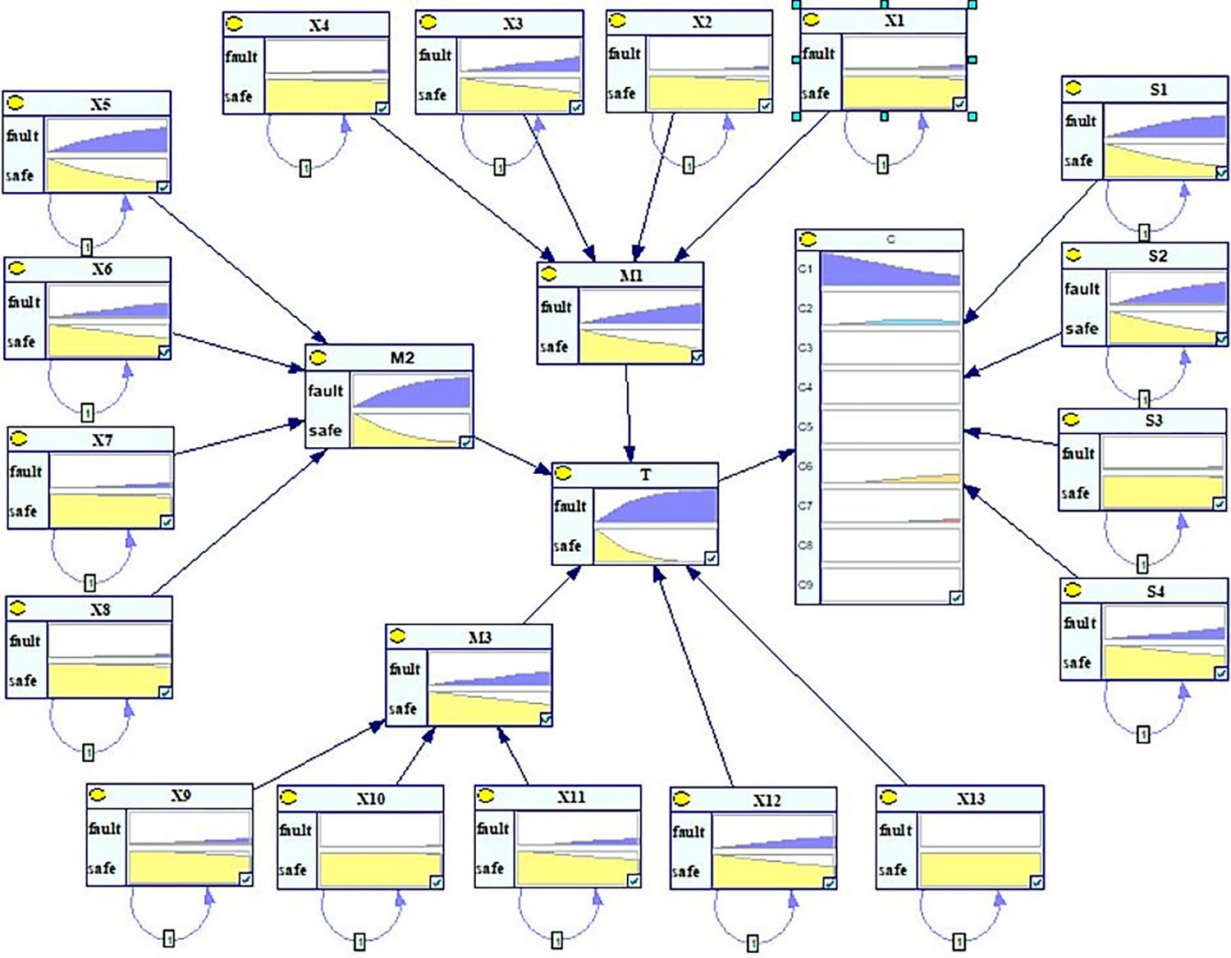
SVDBN model of the coal slurry preparation system.

A program was written in Python according to the data of the prior parameters of the coal slurry preparation system obtained as described above. The results of the GeNIe software reasoning were used to verify the accuracy and reliability of this method. In GeNIe, node prior probability parameters, inter-node conditional probability parameters in the same time slice, and inter-node conditional transition probability parameters were assigned to the risk assessment model. Forward reasoning was carried out on the coal slurry preparation system, and the SVDBN model of the dynamic failure rate of each node in the system running for 2000 h could be obtained, as shown in Fig 10.

**Fig 11:**
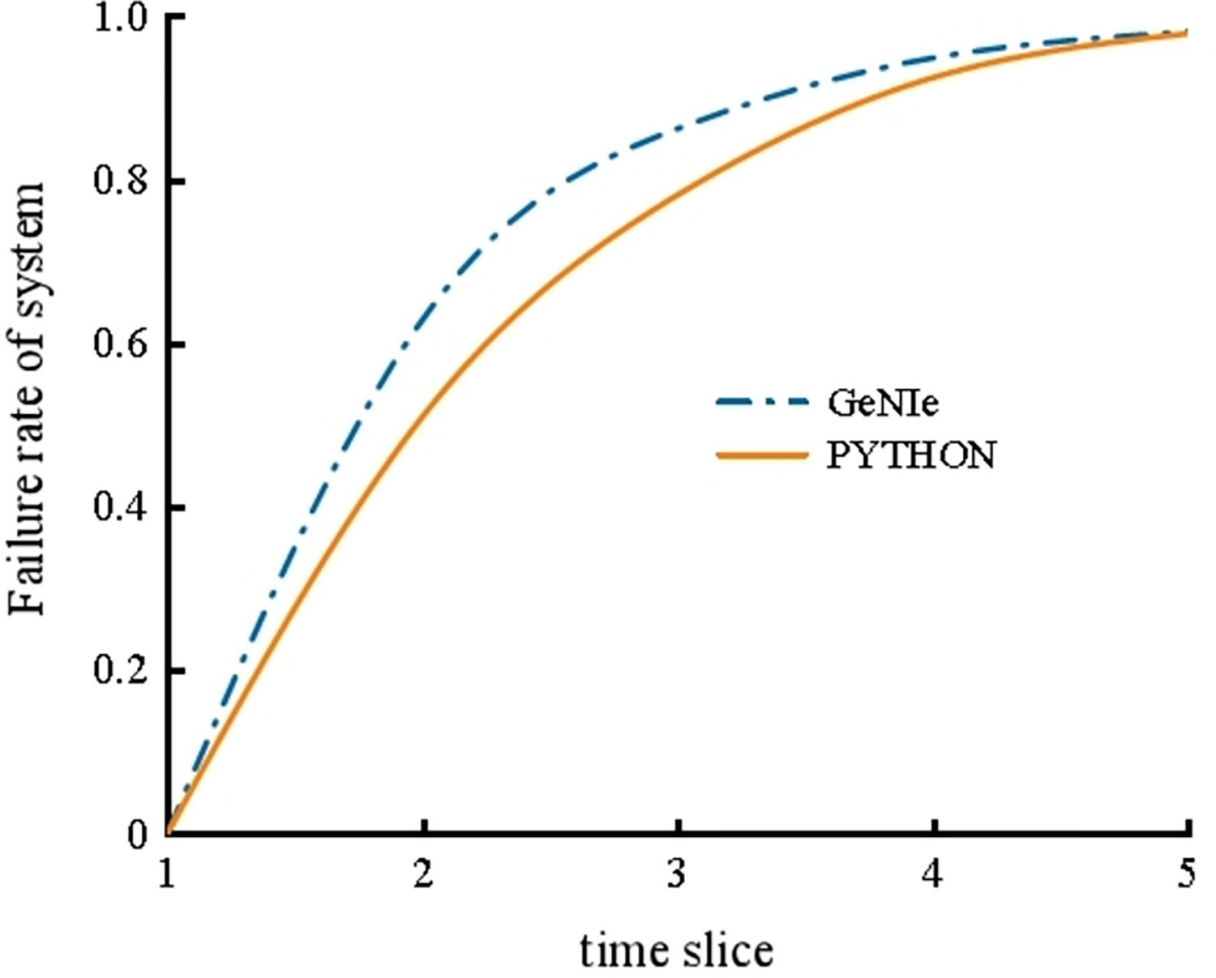
Failure rate of the coal slurry preparation system.

The failure rate curve of the coal slurry preparation system inferred by the two methods is shown in Fig 11. It can be seen that the failure rate of the coal slurry preparation system is close to 1 in the fifth time slice; that is, it runs for nearly 2000 h, and the failure rate gradually increases with increasing time. The reasoning results of the two methods are very similar, which verifies the feasibility of the SVDBN recursive reasoning algorithm described in this paper.

**Fig 12:**
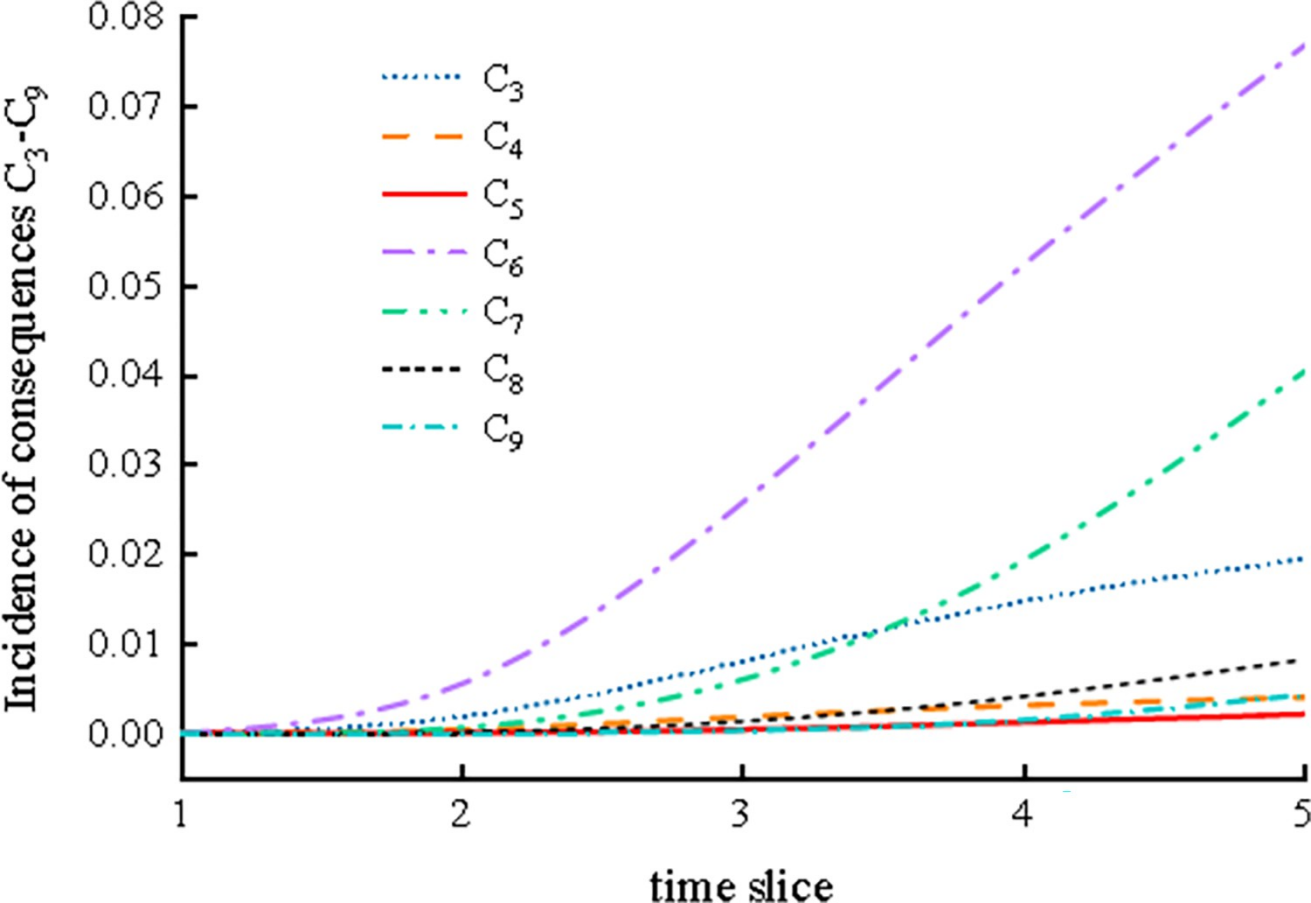
Dynamic trend of the C_3_-C_9_ consequence rate.

At the same time, the possible consequence incidence rate in the system operation process was deduced; this rate influences the dynamic change trend of the C_3_-C_9_ consequence incidence rate of system failure, as shown in Fig 12 (because the C_1_ and C_2_ consequences involve the safe operation of the system, only the consequence incidence rate C_3_-C_9_ is discussed). The dynamic incidence of the consequences caused by the failure of the system in the fifth time slice is shown in Table 4.

**Table 4:** Incidence of the C_3_-C_9_ consequences in the fifth time slice.

Node	Method of this paper	GeNIe	Differentials
C_3_	0.0195	0.0141	0.0054
C_4_	0.004	0.00368	0.00032
C_5_	0.0021	0.00185	0.00025
C_6_	0.0768	0.0705	0.0063
C_7_	0.0404	0.0366	0.0038
C_8_	0.0082	0.00766	0.00054
C_9_	0.0043	0.00371	0.00059

It can be seen that the failure rates of C_3_-C_9_ all show upward trends over time, and the incidence rates of C_3_, C_6_, and C_7_ have the largest change trends. The incidence rates of C_3_, C_6_, and C_7_ are 0.0195, 0.0768, and 0.0404, respectively, which indicates that it is easy for system failure to result in mill slurry running, an abnormal viscosity in the coal slurry, and mill system shutdown. Furthermore, it can be seen from Table 4 that the maximum difference between the probability of consequence occurrence calculated using this reasoning algorithm and GeNIe is no more than 0.007, further confirming the reliability of the SVDBN recursive reasoning algorithm.

### SVDBN diagnostic reasoning of the coal slurry preparation system

**Fig 13:**
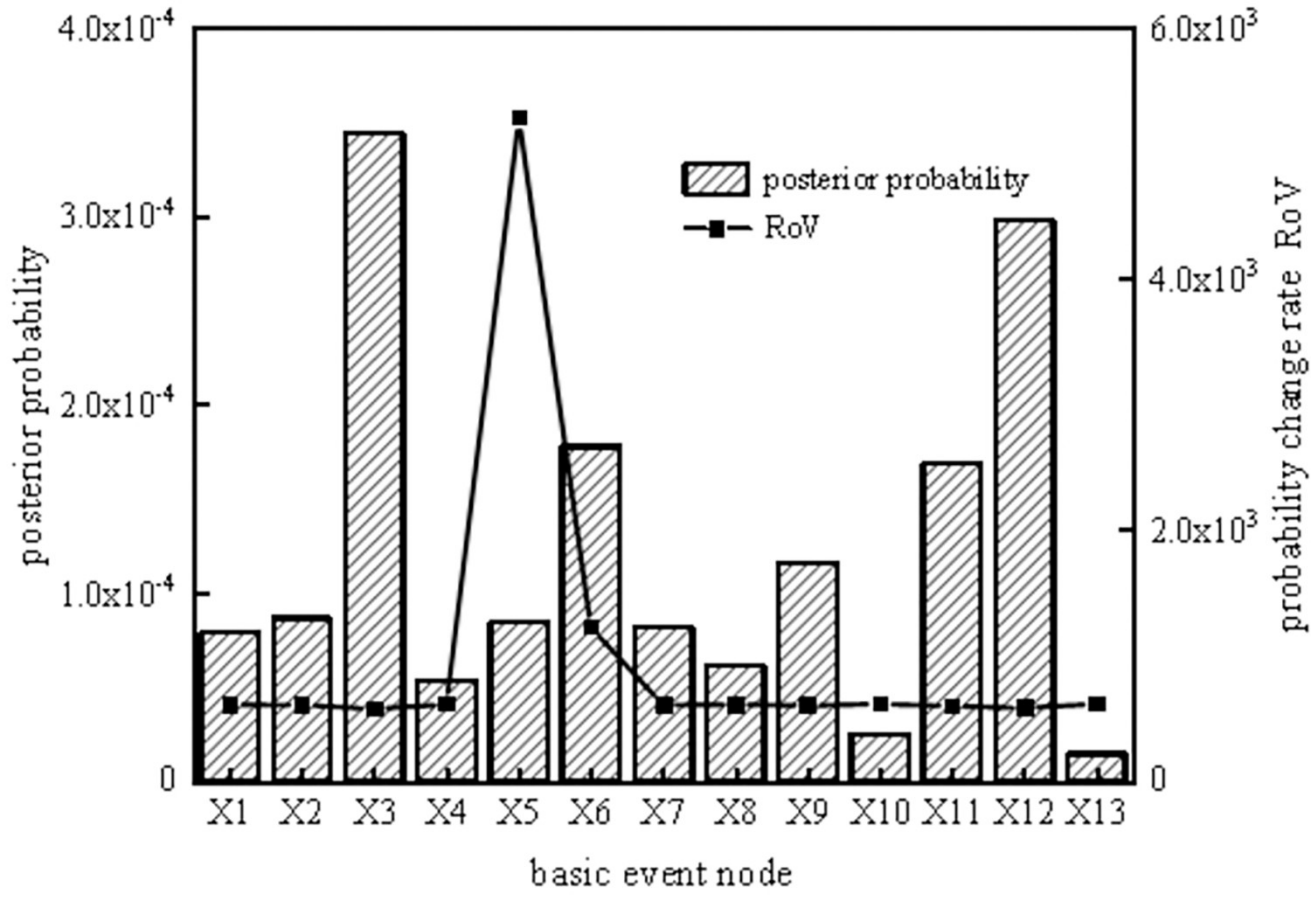
Posterior probability and probability change rate of the second time slice node.

**Fig 14:**
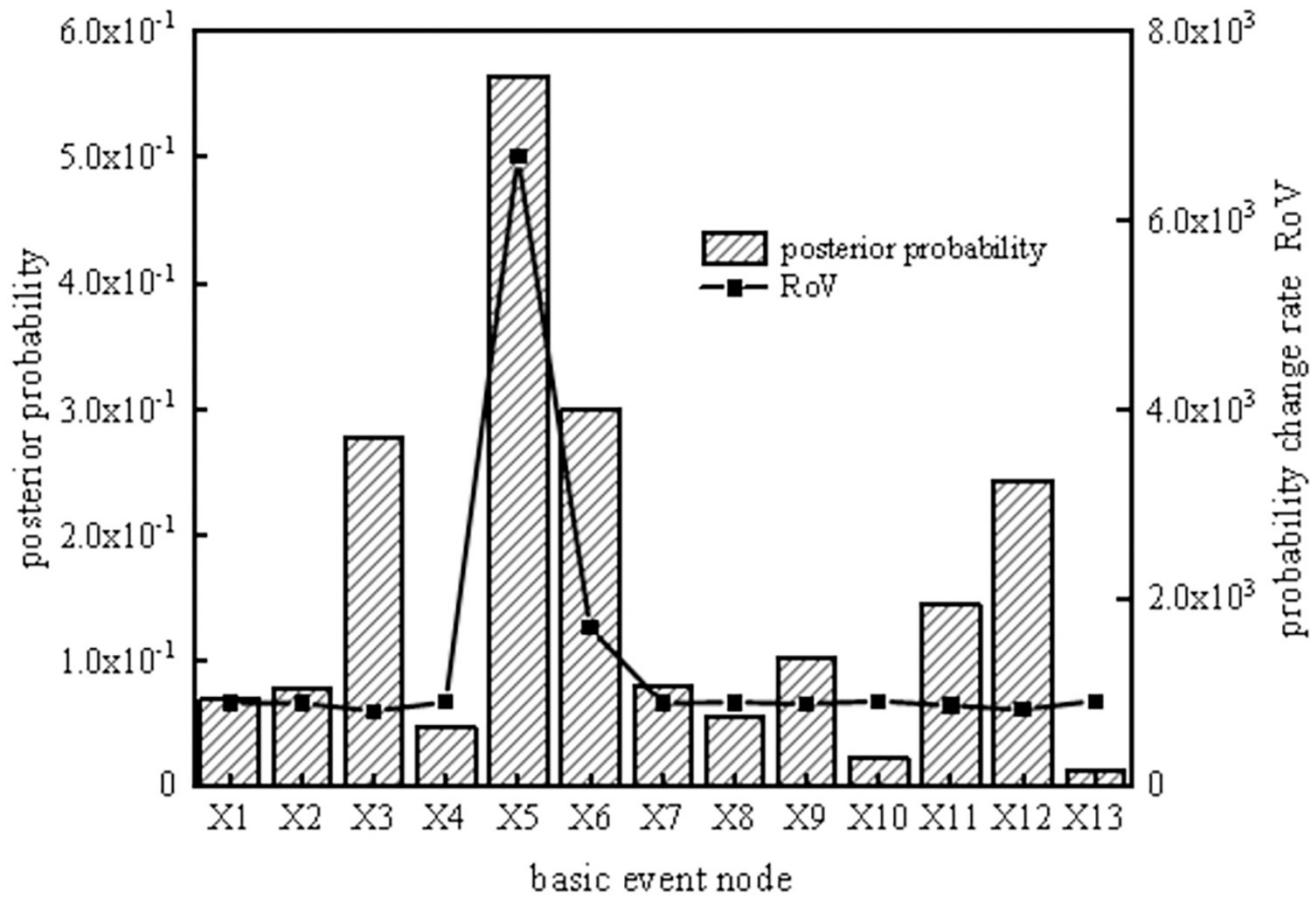
Posterior probability and probability change rate of the third time slice node.

**Fig 15:**
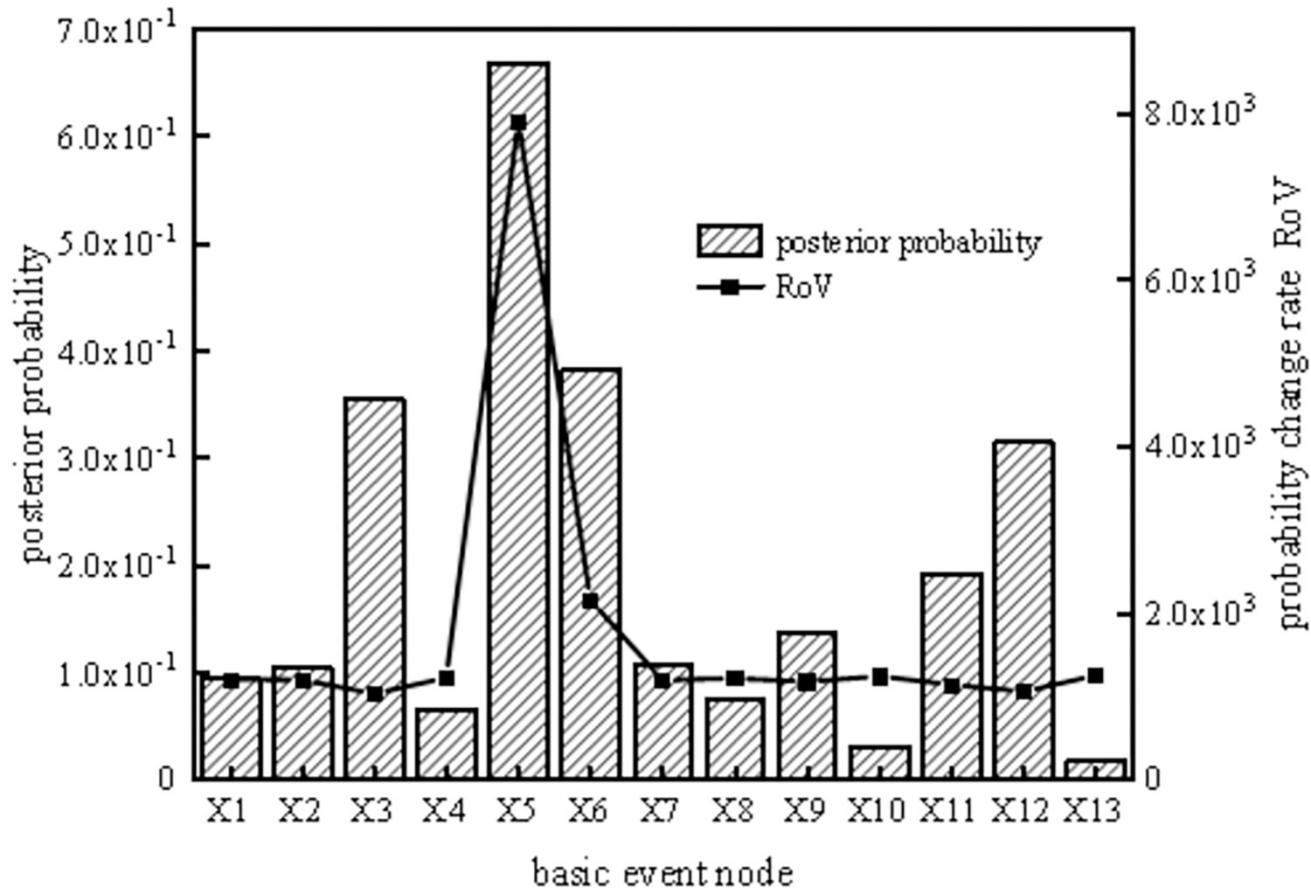
Posterior probability and probability change rate of the fourth time slice node.

**Fig 16:**
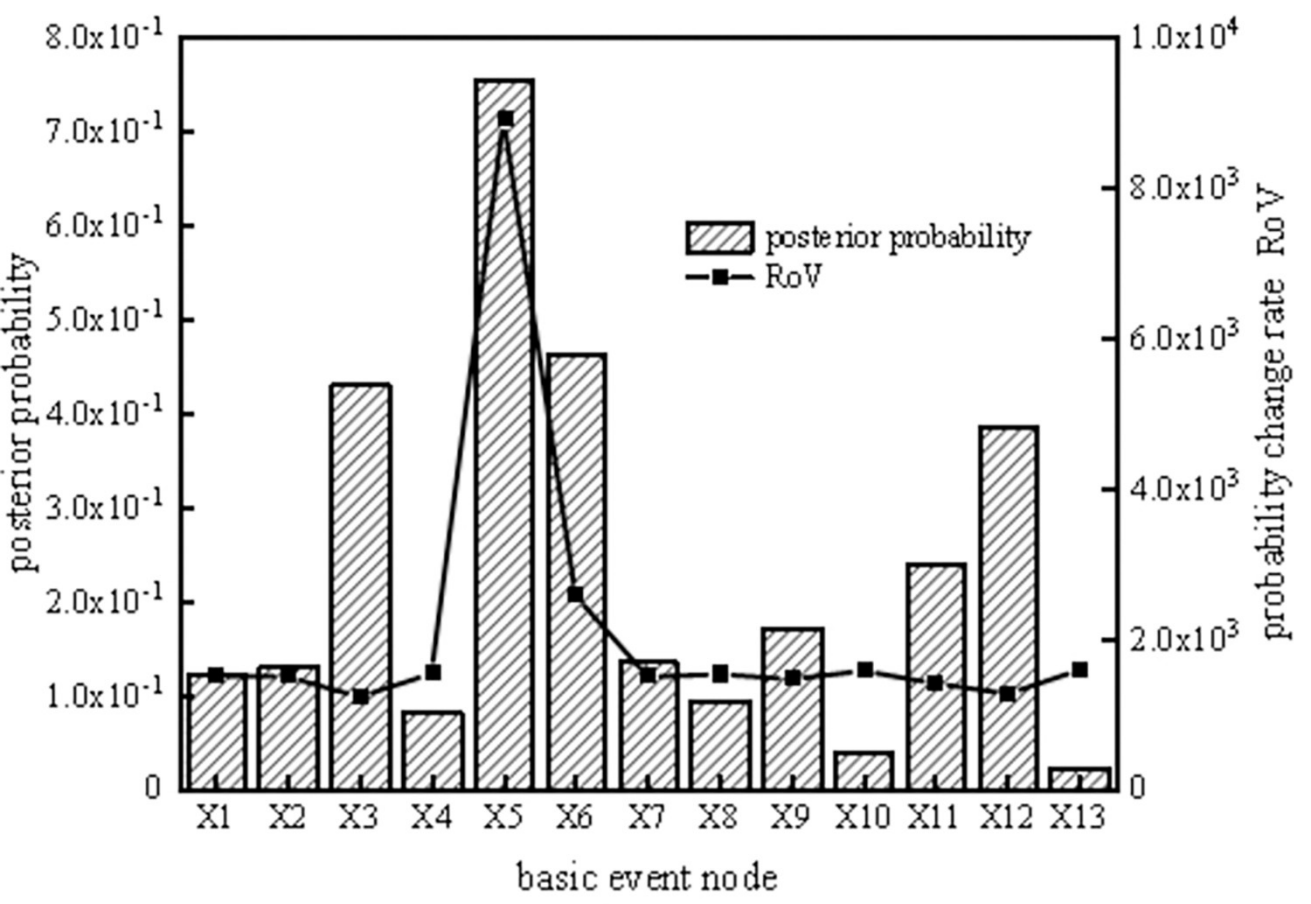
Posterior probability and probability change rate of the fifth time slice node.

According to the reverse reasoning function of the SVDBN, weak links in the system can be diagnosed, enabling the implementation of measures to prevent the occurrence of risks. To analyze the key weak links in the coal slurry preparation system operation cycle, the top event T of the coal slurry preparation system was set as the fault state, and the posterior parameters of the system were obtained under each time slice by taking the system failure as evidence. Then, the ratio of variation (ROV [32]) and posterior probability of each basic event at different time slices can be obtained using the reverse recursive reasoning algorithm of the coal slurry preparation system, as shown in Figs 13–16.

It can be seen from Figs 13–16 that, the larger the ROV, the greater the possibility of node failure. When the system runs for 800 hours (the second time slice), the main weak links are X_3_, X_12_, X_6_, X_11_, X_9_, and X_2_, in order of risk magnitude from large to small. When the system is in the middle period of operation (the third time slice and the fourth time slice), the main weak links are X_5_, X_6_, X_3_, X_12_, X_11_, and X_9_, in order of risk magnitude from large to small. When the system is in the later period of operation (the fifth time slice), the main weak links are X_5_, X_6_, X_3_, X_12_, X_11_, and X_9_, in order of risk magnitude from large to small, which is the same as the results in the middle period of operation. It can be inferred that the weak links of the system are consistent. However, the posterior probability proportion of the nodes in the early stage is different from that in the middle and late stages, so we should focus on X_3_ and X_12_ in the early stage and X_5_ and X_6_ in the middle and late stages.

### Dynamic reliability considering maintenance factors

The results of the SVDBN risk assessment for the coal slurry preparation system without maintenance factors show that the failure rate of the system is close to 1 when it runs for 2000 hours without maintenance. However, if the system is repaired, the fault nodes of the system can be restored to operation, thus reducing the failure rate of the system in each period. Therefore, the influence of maintenance factors on the dynamic reliability of the coal slurry preparation system is considered in the dynamic risk assessment of the variable structure below. The maintenance rate *μ* of the nodes is determined according to the maintenance technology, maintenance cycle, and past maintenance failure records of the coal slurry preparation system. According to Eq (7), the maintenance rate and conditional transition probability of the nodes considering maintenance factors are shown in Table 5.

**Table 5:** Maintenance rate of nodes.

Node	Service percentage *μ*	Conditional transferred probability
X_1_	2.5	0.0821
X_2_	1.67	0.1882
X_3_	1.42	0.2417
X_4_	1.25	0.2865
X_5_	2.5	0.0821
X_6_	1.42	0.2417
X_7_	1.67	0.1882
X_8_	1.42	0.2417
X_9_	2.5	0.0821
X_10_	1.67	0.1882
X_11_	1.24	0.2894
X_12_	1.25	0.2865
X_13_	1.25	0.2865
S_1_	1.42	0.2417
S_2_	2.5	0.0821
S_3_	1.25	0.2865
S_4_	1.25	0.2865

Taking X_1_ as an example, the conditional transition probability of the node considering maintenance factors is shown in Table 6.

**Table 6:** Conditional transition probability of nodes considering maintenance factors.

X_1_	Fault	Safe
fault	0.0821	0.0312
safe	0.9179	0.9688

**Fig 17:**
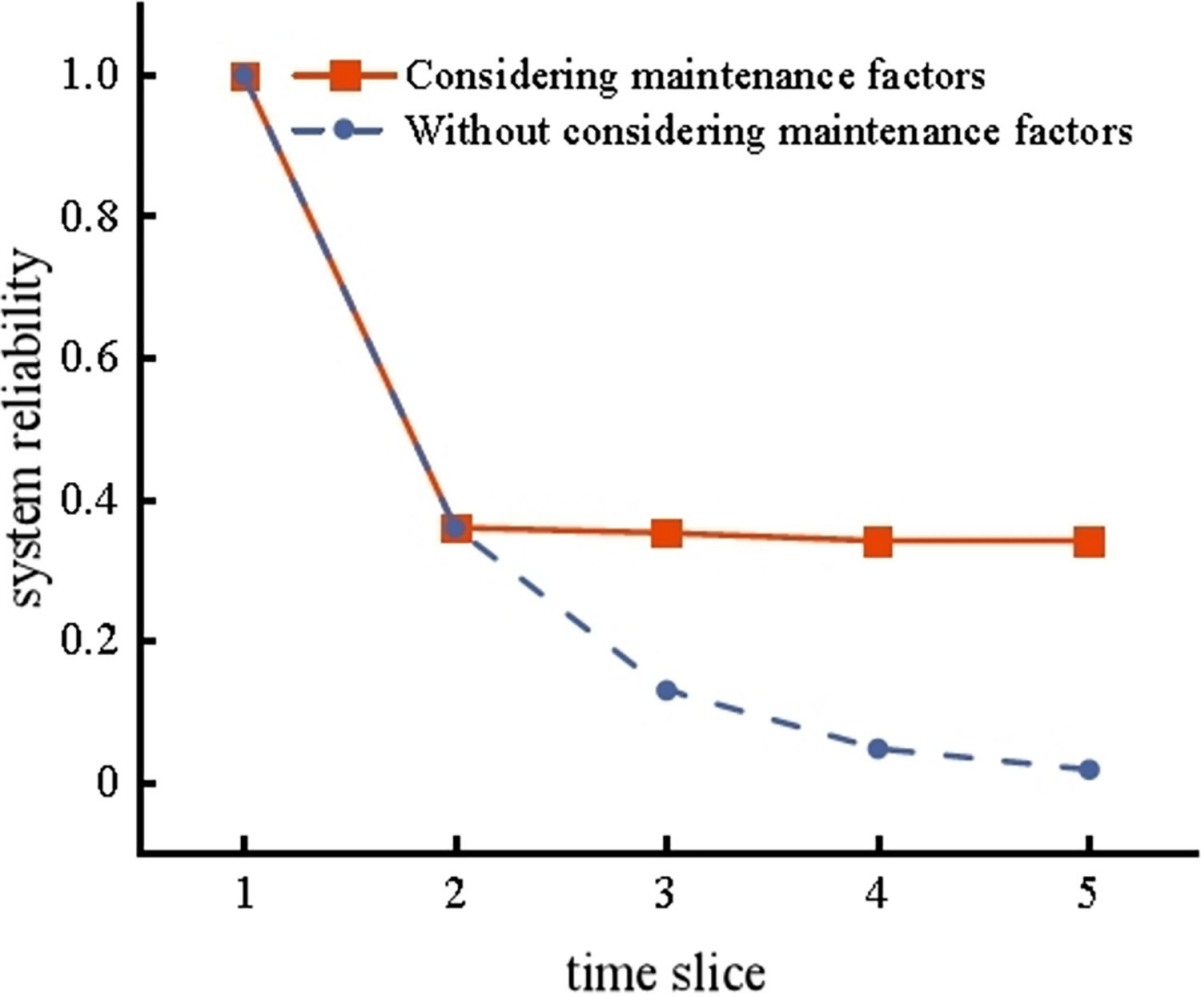
Reliability of coal slurry preparation system with and without maintenance factors.

According to the node maintenance rate and probability of conditional transition, SVDBN forward reasoning was performed on the coal slurry preparation system, and the reliability of the coal slurry preparation system T could be obtained under the conditions of considering or not considering maintenance factors, as shown in Fig 17.

It can be seen from Fig 17 that the reliability of the coal slurry preparation system considering maintenance factors is much higher than that without considering maintenance factors, and the dynamic reliability of the system will gradually tend to a stable value of 0.34 during operation. It can be seen that maintenance factors, such as maintenance technology and maintenance operation, improve the reliability of the system. Therefore, maintenance work is essential to improve the reliability of the system and reduce risk.

**Fig 18:**
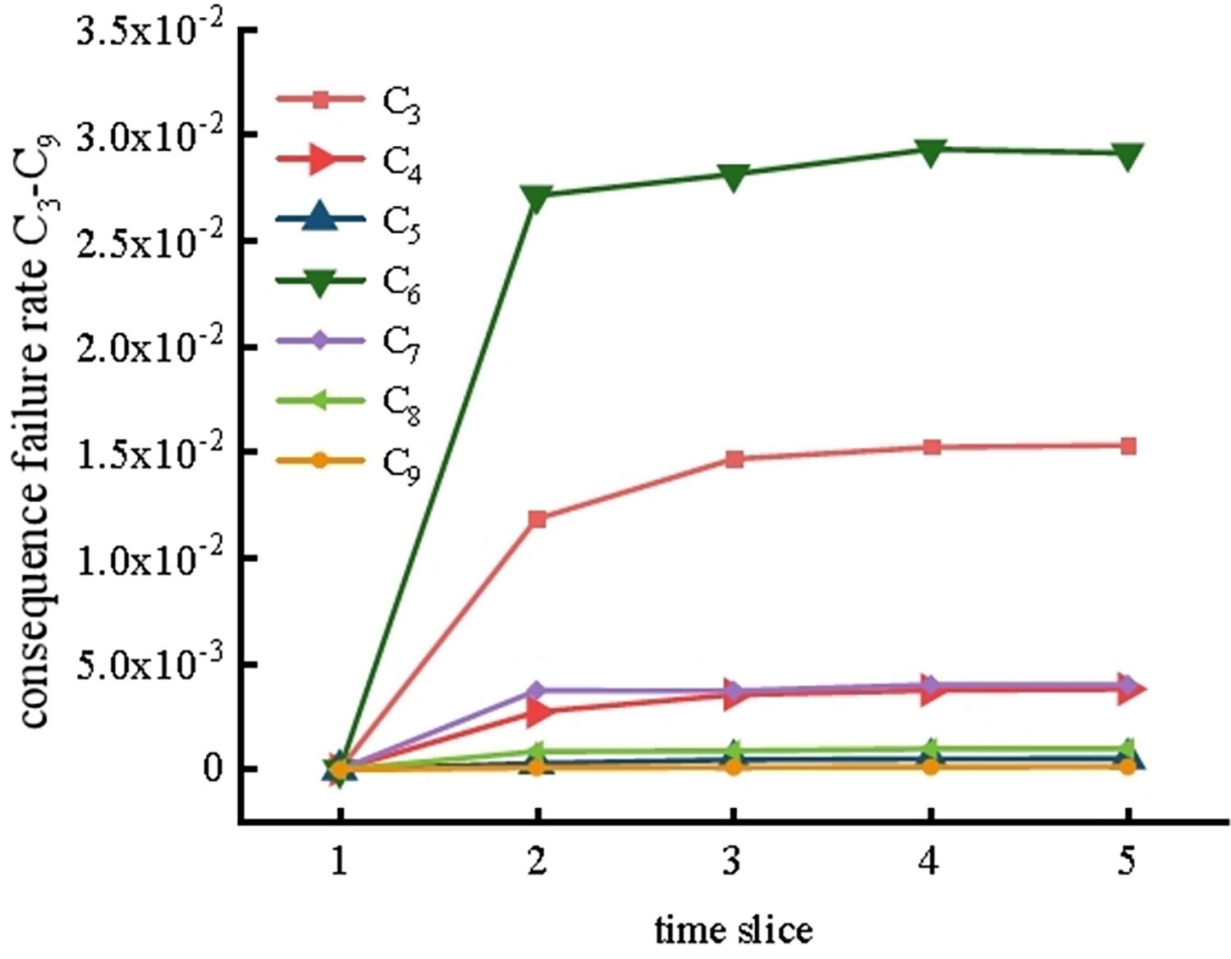
Consequential failure rates for C_3_-C_9_ when maintenance factors are considered.

The forward reasoning of the SVDBN can also obtain the dynamic consequence rate of the coal slurry preparation system when considering maintenance factors, as shown in Fig 18. Therefore, it can be seen that, when maintenance factors are considered in the system, the dynamic incidence of consequences C_3_-C_9_ in a dangerous state decreases under the influence of maintenance and remains in a stable probability range. However, the incidence of C_3_ and C_6_ consequences is higher than that of other consequences, which indicates that it is easy for system failure to result in mill slurry running, an abnormal viscosity in the coal slurry, and mill system shutdown.

Similarly, the reverse reasoning of the SVDBN is performed on the coal slurry preparation system according to the maintenance rate of the node and the probability of conditional transition, and the posterior probability of the nodes of each basic event can be obtained at different time slices when considering maintenance factors, as shown in Table 7.

**Table 7:** Posterior probability of basic events when maintenance factors are considered.

Node	Time slice 1	Time slice 2	Time slice 3	Time slice 4	Time slice 5
X_1_	0.0495	0.0487	0.0505	0.0498	0.0498
X_2_	0.0543	0.0533	0.0608	0.0610	0.0612
X_3_	0.2151	0.2006	0.2206	0.2194	0.2197
X_4_	0.0335	0.0330	0.0413	0.0429	0.0435
X_5_	0.0529	0.4477	0.3518	0.3642	0.3605
X_6_	0.1113	0.2189	0.2383	0.2364	0.2367
X_7_	0.0558	0.0548	0.0624	0.0626	0.0628
X_8_	0.0383	0.0378	0.0455	0.0464	0.0468
X_9_	0.0725	0.0707	0.0725	0.0713	0.0713
X_10_	0.0160	0.0159	0.0185	0.0187	0.0188
X_11_	0.1057	0.1020	0.1234	0.1262	0.1274
X_12_	0.1864	0.1753	0.2034	0.2051	0.2060
X_13_	0.0093	0.0092	0.0117	0.0122	0.0124

**Fig 19:**
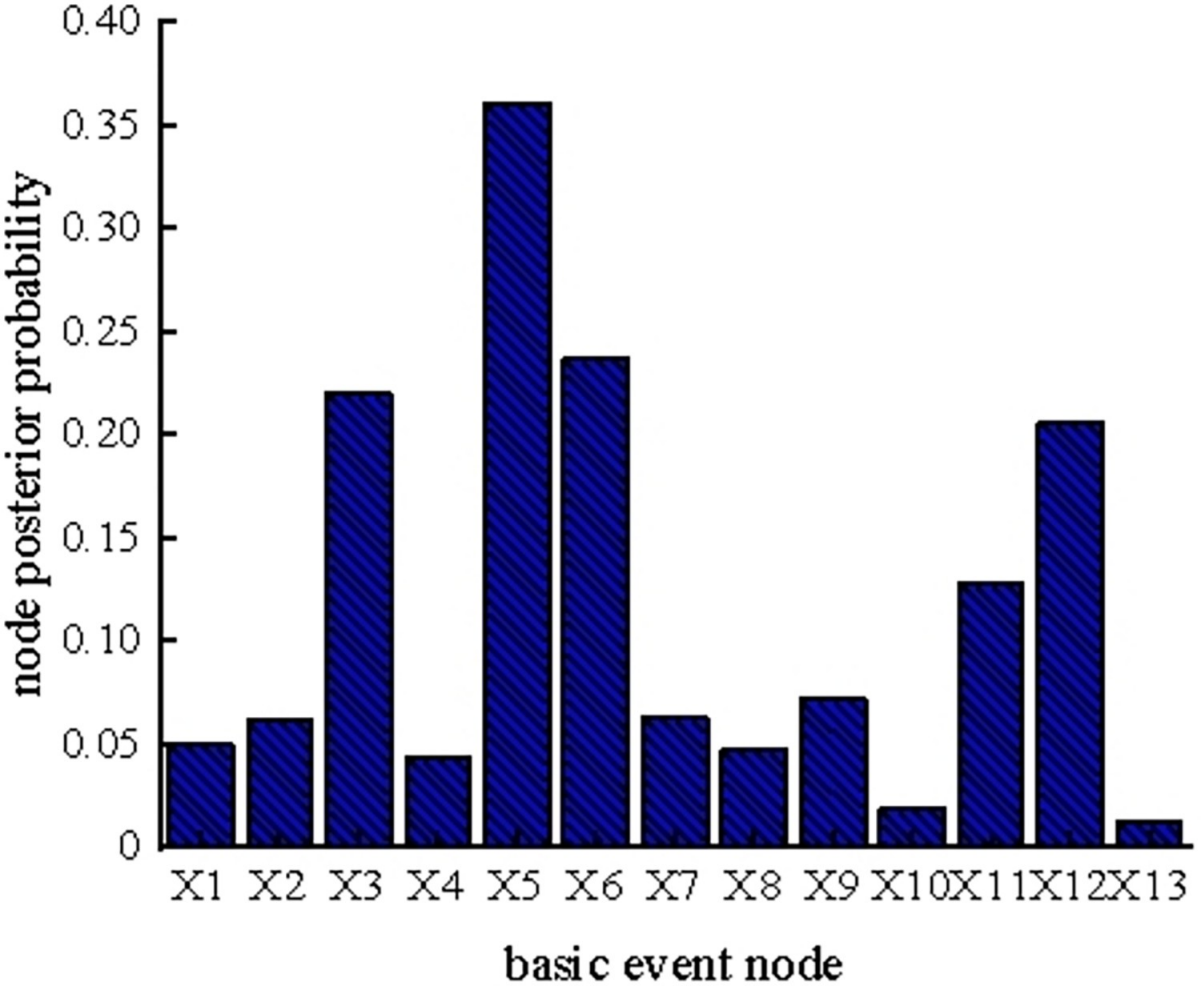
Posterior probability of nodes considering maintenance factors in the fifth time slice.

The above results show that the posterior probabilities of the basic event nodes under different maintenance conditions are relatively stable in different time slices, and the posterior probabilities of each node are maintained within a certain threshold range. In particular, the posterior probability of the basic event nodes when considering maintenance in the fifth time slice is shown in Fig 19.

It can be seen from the figure that the first six nodes with a higher posterior probability value considering maintenance factors in the fifth time slice are X_3_, X_5_, X_6_, X_9_, X_11_, and X_12_. These six weak links are consistent with the weak links of reasoning without considering maintenance factors, which not only verifies the feasibility and accuracy of the reasoning method, but also shows that the above node events are still the key links in the system after a maintenance operation. Companies can improve the maintenance rate of weak links by optimizing their maintenance strategy and maintenance technology, consequently enhancing the reliability of the coal slurry preparation system.

## Conclusions

The BT model can combine the fault tree and event tree, which are widely used in safety evaluation, and fully characterize the logical relationship of risk factors in the accident chain. Accidents can be effectively prevented by identifying hazard sources, discriminating risk factors, setting safety barriers, and taking control measures to prevent and reduce risks. The BT model was used to qualitatively analyze the key equipment of the coal chemical industry. The qualitative risk assessment model for coal slurry preparation system failure can be systematically constructed from the cause to the result of events.

Taking into account the time-varying and unstable operation of the system equipment in the coal gasification process, the SVDBN was used for the dynamic risk assessment of coal slurry preparation system faults in an unsteady state, which could effectively improve the accuracy of dynamic risk assessment results. Under the causal reasoning of the SVDBN, the dynamic trend of accident risk can be intuitively predicted. The key factors leading to the failure of the coal slurry preparation system in different periods were compared on the basis of diagnostic reasoning, which can help enterprises to optimize their maintenance plans and formulate preventive measures to ensure the long-term steady-state operation of the coal slurry preparation process.

Based on the SVDBN recursive inference algorithm, the SVDBN dynamic risk analysis of the coal slurry preparation system was implemented in Python. The weak nodes in the system can be identified according to the reverse inference function of the SVDBN, and the failure rate can be reduced by shortening the maintenance period of the weak links. The safety of the system can also be enhanced by improving the performance of the weak links, prolonging the service life, and increasing the protective devices, which provides the basis for the safety process design, safety management, and emergency management objectives of the system.

## Supporting information

S1 File Dynamic risk assessment of a coal slurry preparation system based on the structure-variable Dynamic Bayesian Network. (DOC)
