# Supplementary material for: Dynamic risk assessment of a coal slurry preparation system based on the structure-variable Dynamic Bayesian Network
Source: PLoS One. 2024 May 21;19(5):e0302044. doi: 10.1371/journal.pone.0302044 (PMC11108168; doi:10.1371/journal.pone.0302044)
Supplement: S1 File — (DOC) [file pone.0302044.s001.doc]

**Dynamic risk assessment of a coal slurry preparation system based on the** **Structure-variable Dynamic Bayesian Network**

**Supplementary Materials**

**Table S1 Failure rate of the coal slurry preparation system, presented in Fig 11.**

| Time slice | GeNIe | Python |
| --- | --- | --- |
| 1 | 0.00129 | 0.0017 |
| 2 | 0.63 | 0.5112 |
| 3 | 0.86292 | 0.7822 |
| 4 | 0.94922 | 0.9252 |
| 5 | 0.98119 | 0.9791 |

Table S2 Dynamic trend of the C_3_-C_9_ consequence rate, presented in Fig 12.

| Time slice | C_3_ | C_4_ | C_5_ | C_6_ | C_7_ | C_8_ | C_9_ |
| --- | --- | --- | --- | --- | --- | --- | --- |
| 1 | 0 | 0 | 0 | 0 | 0 | 0 | 0 |
| 2 | 0.0019 | 4.00E-04 | 0 | 0.0055 | 6.00E-04 | 1.00E-04 | 0 |
| 3 | 0.0081 | 0.0018 | 4.00E-04 | 0.0258 | 0.006 | 0.0013 | 3.00E-04 |
| 4 | 0.0148 | 0.0031 | 0.0012 | 0.0523 | 0.0193 | 0.0041 | 0.0015 |
| 5 | 0.0195 | 0.004 | 0.0021 | 0.0768 | 0.0404 | 0.0082 | 0.0043 |

**Table S3 Posterior probability and probability change rate of the second time slice node, presented in Fig 13.**

| Time slice | Posterior probability | Probability change rate RoV |
| --- | --- | --- |
| X1 | 7.92E-05 | 614.4522 |
| X2 | 8.69E-05 | 6.13E+02 |
| X3 | 3.44E-04 | 582.7589 |
| X4 | 5.35E-05 | 617.3005 |
| X5 | 8.46E-05 | 5288.774 |
| X6 | 1.78E-04 | 1228.939 |
| X7 | 8.21E-05 | 612.8966 |
| X8 | 6.13E-05 | 616.3321 |
| X9 | 1.16E-04 | 609.6343 |
| X10 | 2.56E-05 | 620.7654 |
| X11 | 1.69E-04 | 603.243 |
| X12 | 2.98E-04 | 588.0517 |
| X13 | 1.48E-05 | 622.1468 |

**Table S4 Posterior probability and probability change rate of the third time slice node, presented in Fig 14.**

| Time slice | Posterior probability | Probability change rate RoV |
| --- | --- | --- |
| X1 | 7.06E-02 | 890.1421 |
| X2 | 7.72E-02 | 8.87E+02 |
| X3 | 2.76E-01 | 802.4583 |
| X4 | 4.81E-02 | 898.8252 |
| X5 | 5.64E-01 | 6670.307 |
| X6 | 3.00E-01 | 1682.289 |
| X7 | 7.92E-02 | 886.1328 |
| X8 | 5.50E-02 | 896.0315 |
| X9 | 1.02E-01 | 876.8154 |
| X10 | 2.33E-02 | 908.8902 |
| X11 | 1.45E-01 | 858.7287 |
| X12 | 2.44E-01 | 816.753 |
| X13 | 1.35E-02 | 912.8762 |

Table S5 Posterior probability and probability change rate of the fourth time slice node, presented in Fig 15.

| Time slice | Posterior probability | Probability change rate RoV |
| --- | --- | --- |
| X1 | 9.52E-02 | 1200.713 |
| X2 | 1.04E-01 | 1.19E+03 |
| X3 | 3.55E-01 | 1030.432 |
| X4 | 6.53E-02 | 1218.546 |
| X5 | 6.69E-01 | 7901.643 |
| X6 | 3.82E-01 | 2147.482 |
| X7 | 1.07E-01 | 1192.969 |
| X8 | 7.44E-02 | 1212.901 |
| X9 | 1.36E-01 | 1174.352 |
| X10 | 3.17E-02 | 1238.996 |
| X11 | 1.93E-01 | 1138.571 |
| X12 | 3.15E-01 | 1057.446 |
| X13 | 1.85E-02 | 1247.103 |

**Table S6 Posterior probability and probability change rate of the fifth time slice node, presented in Fig 16.**

| Time slice | Posterior probability | Probability change rate RoV |
| --- | --- | --- |
| X1 | 1.21E-01 | 1528.761 |
| X2 | 1.32E-01 | 1.52E+03 |
| X3 | 4.31E-01 | 1250.787 |
| X4 | 8.35E-02 | 1559.227 |
| X5 | 7.54E-01 | 8914.007 |
| X6 | 4.62E-01 | 2592.036 |
| X7 | 1.35E-01 | 1515.969 |
| X8 | 9.51E-02 | 1549.649 |
| X9 | 1.72E-01 | 1484.743 |
| X10 | 4.08E-02 | 1594.102 |
| X11 | 2.41E-01 | 1425.328 |
| X12 | 3.86E-01 | 1293.669 |
| X13 | 2.38E-02 | 1607.97 |

Table S7 Reliability of coal slurry preparation system with and without maintenance factor, presented in Fig 17.

| Time slice | Considering maintenance factors | Without considering maintenance factors |
| --- | --- | --- |
| 1 | 0.9984 | 0.9984 |
| 2 | 0.35884 | 0.35834 |
| 3 | 0.35131 | 0.12862 |
| 4 | 0.34017 | 0.04616 |
| 5 | 0.34026 | 0.01657 |

**Table S8 Consequential failure rate C_3_-C_9_ when maintenance factors are considered, presented in Fig 18.**

| Time slice | C_3_ | C_4_ | C_5_ | C_6_ | C_7_ | C_8_ | C_9_ |
| --- | --- | --- | --- | --- | --- | --- | --- |
| 1 | 5.42E-11 | 1.30E-11 | 3.37E-15 | 1.44E-10 | 3.73E-14 | 8.95E-15 | 2.32E-18 |
| 2 | 0.01186 | 0.00273 | 2.98E-04 | 0.02714 | 0.00375 | 8.65E-04 | 9.45E-05 |
| 3 | 0.01469 | 0.00354 | 4.69E-04 | 0.02816 | 0.00373 | 9.00E-04 | 1.19E-04 |
| 4 | 0.01524 | 0.00376 | 5.14E-04 | 0.02933 | 0.00402 | 9.90E-04 | 1.35E-04 |
| 5 | 0.01533 | 0.00381 | 5.25E-04 | 0.02916 | 0.00402 | 9.98E-04 | 1.37E-04 |

**Table S9 Posterior probability of nodes considering maintenance factors in the fifth time slice, presented in Fig 19.**

| Basic event node | Node posterior probability |
| --- | --- |
| X1 | 0.04983 |
| X2 | 0.06118 |
| X3 | 0.21968 |
| X4 | 0.04347 |
| X5 | 0.36048 |
| X6 | 0.23667 |
| X7 | 0.06277 |
| X8 | 0.04681 |
| X9 | 0.07135 |
| X10 | 0.01877 |
| X11 | 0.12736 |
| X12 | 0.20605 |
| X13 | 0.01236 |
